# Successful surgical management of pace-marker induced infective endocarditis under the guidance of real-time three-dimensional trans-esophageal echocardiogram

**DOI:** 10.1186/1749-8090-9-96

**Published:** 2014-05-31

**Authors:** Jun Gu, Da Zhu, Eryong Zhang

**Affiliations:** 1 Department of Cardiovascular Surgery, Sichuan University, West China Hospital, Cheng du, Si chuan, PR China

**Keywords:** Real-time three-dimensional trans-esophageal echocardiogram, Pacemaker endocarditis, Vegetation

## Abstract

The infection of cardiac implantable electronic device is a serious and potentially lethal complication. Accurate preoperative evaluation of location of vegetation, cardiac valve pathology is of paramount important. We reported a case of 71 year-old male patient who suffered from pacemaker endocarditis was given suitable surgical treatment under the guidance of real-time three-dimensional trans-esophageal echocardiogram.

## Background

The infection of cardiac implantable electronic device is a serious and potentially lethal complication especially in older patients with associated comorbid conditions. Accurate preoperative evaluation of location of vegetation, cardiac valve pathology is of paramount important. Real-time 3-dimensional trans-esophageal echocardiography (RT3D TEE) is able to demonstrate precise relationships among various anatomical structures and visualize intra-cardiac catheters, vegetation as well as the valve morphology while without artifact associated with TTE and conventional TEE which would largely facilitate surgical strategy making. We then report a case of successful management of pacemaker induced infective endocarditis under the guidance of RT3D TEE.

## Case presentation

**Figure 1:**
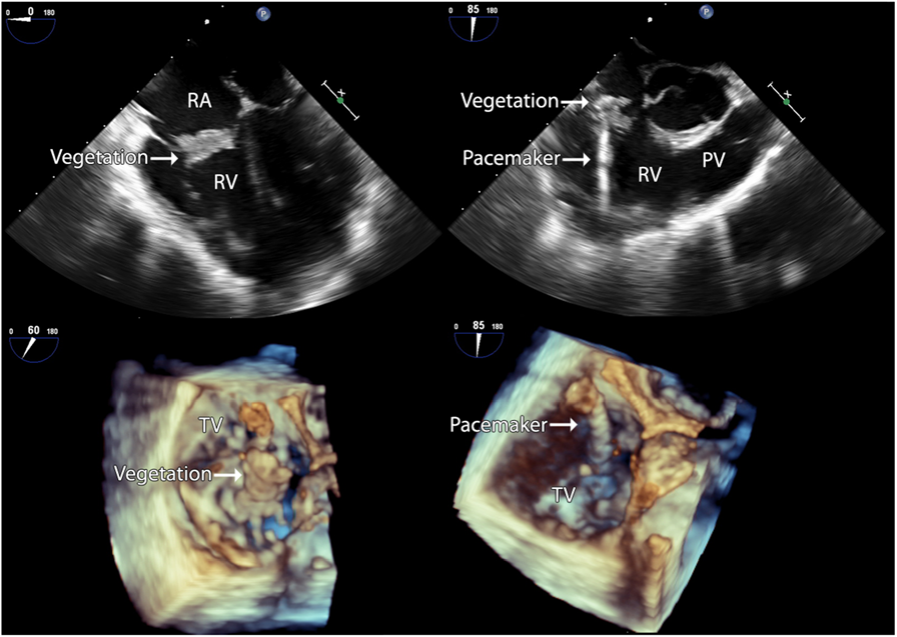
**TEE image of this patient with pacemaker induced endocarditis of tricuspid valve.** Upper panel: Mid-esophageal four chamber and aortic valve short axis views showed a mass attaching to the tricuspid valve. Lower panel: RT3D TEE confirmed highly mobile vegetation attached to posterior and septal leaflet of the tricuspid valve which was originated from pacemaker lead. During systolic phase (Left panel) the vegetation was protruding into right atrium, during diastolic phase (Right panel) the vegetation was moving back to right ventricle with tricuspid valve indicating the vegetation was attach to the tricuspid valve.

**Figure 2:**
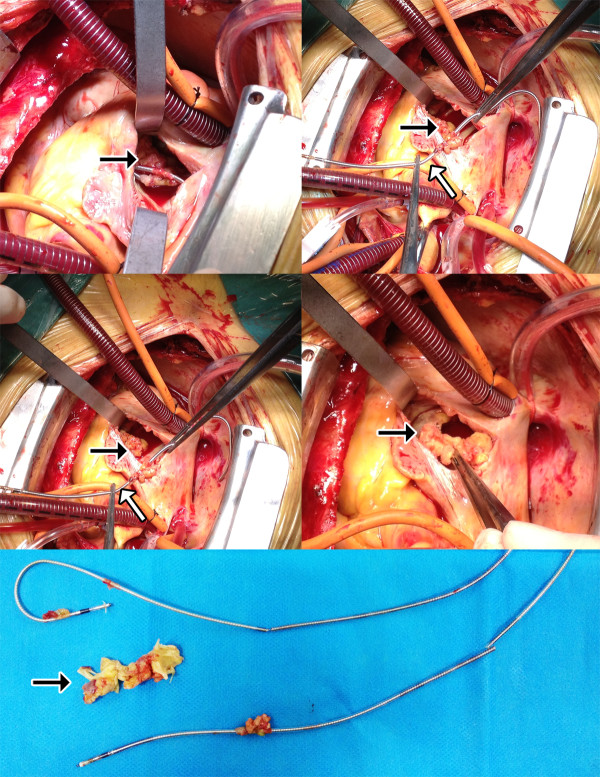
**Intraoperative image.** Intraoperative image showed a giant vegetation attaching to both the tricuspid valve originating from pacemaker lead causing perforation of the tricuspid valve which is identical to RT3D TEE image (Black arrow show).

A 71 year-old male patient of Han nationality was admitted to the emergency department with 4-weeks history of recurrent fever, cough and hemoptysis which were refractory to broad spectrum antibiotics. He had history of dual-chamber pacemaker implantation due to sick sinus syndrome. On admission, computed tomography of the chest demonstrated bilateral pulmonary infiltrate. Multiple blood cultures confirmed positive for staphylococcus aureus. Trans-thoracic echocardiogram (TTE) revealed the pacemaker in the right ventricular and a giant vegetation sized 16*10 mm in the tricuspid valve causing moderate insufficiency, however, due to poor image quality, relationship between pacemaker lead and the vegetation could not be identified. Trans-esophageal echocardiogram (TEE) was then utilized for further evaluation which confirmed not only a large vegetation attaching to the tricuspid valve causing tricuspid valve insufficiency but also no thickening, adhesion or limited opening of the tricuspid valve (Figure 1 - upper panel). Further RT3D TEE confirmed a highly mobile vegetation sized 17*11*8 mm attach to posterior as well as septal leaflet of the tricuspid valve which was originated from pacemaker lead. (Figure 1 - lower panel). This patient was then underwent open heart surgical procedure done through the right atrium with cardio-pulmonary bypass and cardiac arrest. A giant vegetation originated from pace-marker lead while attaching to posterior as well as septal leaflet of the tricuspid valve was noted during intraoperative exploration which was identical to preoperative 3D TEE (Figure 2). As for this patient, extraction of the pace-marker lead, tricuspid valve replacement with bio-prosthetic valve, as well as epicardial temporary pacemaker implantation was done due to massive damage of the valve leaflet. The patient recovered fully on antibiotic administration and was discharged afebrile 4 weeks after surgery and remained stable without any sign of recurrence 3 months after discharge.

## Discussion

Infection is a serious complication of permanent pacemaker implantation. Reported incidence rates of pacemaker infection ranged from 0.13% to 19.9% [1]. Although most infections have been limited to the subcutaneous pocket, frank pacemaker endocarditis still accounts for approximately 10% of total cases with mortality rates as high as 31% to 66% if the infected device is not removed [2,3]. Complete device removal combine with antimicrobial therapy is the only effective method which could decrease the mortality to 18% or less [4]. Study had shown that both percutaneous lead-removal and open cardiovascular surgery for complete device removal were two effective methods as for treatment of pacemaker endocarditis [5]. Compare with percutaneous lead-removal, open heart surgical removal is advocated for patient with cardiac valve evolvement and lead with large vegetation due to the possibility of dislodging emboli.

Precise delineation of pacemaker infection such as identifying the vegetation location, pacemaker lead position as well as valve pathology allows therapeutic medical and surgical treatment decisions. This process may be challenging by routine trans-thoracic echocardiography due to reverberation artifact produced by the intra-cardiac catheter [6]. Cardiac magnetic resonance is also currently not a feasible imaging option as for patients with pacemaker. In contrast, real-time 3-dimensional trans-esophageal echocardiography is able to visualize intra-cardiac catheters, including catheter tip and demonstrate precise relationships between these device and important intra-cardiac structure while without artifact associated with TTE. As shown in this case, 3D-TEE clearly delineation the location and relationship to valve structure of vegetation which was identical to direct surgical vision. Due to destruction of the tricuspid valve and giant vegetation formation, open heart surgery with complete device removal and valve replacement is the optional method as for this patient.

## Conclusion

2D echocardiography could be easily interfered by artifact induced by artificial implantable device such as pacemaker wire. In contrast, RT3D TEE can provide “surgical view” class which could largely facilated the treatment strategy.

## Patient informed consent

Written informed consent was obtained from the patient for publication of this Case report and any accompanying images. A copy of the written consent is available for review by the Editor-in-Chief of this journal.

## Abbreviations

TTE: Trans-thoracic echocardiography, TEE, Trans-esophageal echocardiography, RT3D TEE, Real-time three-dimensional trans-esophageal echocardiogram.

## Competing interests

All the authors declare that they have no competing interests.

## Authors’ contributions

In the following we specify the individual contributions of authors to the manuscript. All surgical procedures were performed by EYZ, JG and DZ. JG and DZ managed the perioperative period of the patient. JG prepared the manuscript and EYZ made final approval. All authors read and approved the final manuscript.
